# Supplementary material for: “I Carry the Trauma and Can Vividly Remember”: Mental Health Impacts of the COVID-19 Pandemic on Frontline Health Care Workers in South Africa
Source: Int J Environ Res Public Health. 2023 Jan 29;20(3):2365. doi: 10.3390/ijerph20032365 (PMC9914980; doi:10.3390/ijerph20032365)
Supplement: Supplementary file 1 [file ijerph-20-02365-s001.zip › ijerph-2059821-supplementary.pdf]

Supplementary materials

Table S1: Interview guide used during interviews with FHCWs

1. **To understand how COVID-19 has impacted on the lives of front-line workers who provided services during the pandemic of COVID-19.**
    - Can you please tell me about the work you, department or ward, and how long you have been there?
    - Can you tell me about your job since the outbreak of COVID-19, has anything changed, if yes, how? (Probe about workload, and precautionary measures).
    - Can you comment on the support and other enablers that are provided for you to do your job now during COVID-19 pandemic?
    - What can be improved?
2. **To explore how being in the frontline during the pandemic influenced dynamics in their relationships with partners and children.**
    - Can you tell me about your relationship with your family since the outbreak? (Probe about time spent at home or not).
    - Can you tell me about your relationship with your children, comparing it before and during the outbreak?
    - Can you tell me about your relationship with your partner/spouse, comparing it before and during the outbreak? (Probe: has it been affected in any way since the outbreak).
    - Please share with me instances of tensions and conflict with your significant others resulting from you working since the outbreak of Covid-19.
        i. Probe, if they indicate experiences of conflict, ask to describe the kind of conflict and tension (was it verbal or physical)
        ii. How was the conflict or tension resolved?
3. **To understand the perceived impact of COVID-19 on mental health of frontline workers**
    - What are your worries and concerns related to your job since the outbreak? (Probe for worries specific to work).
    - What are your worries and concerns related to your family since the outbreak? (Probe for worries specific to family, children, spouse).
    - Can you comment on your physical well-being since the outbreak, has it changed in any way? (Probe how they are affected, and how they feel).
    - Can you comment on your emotional well-being since the outbreak, has it changed in any way? (Probe how they are affected, and how they feel).
    - Please describe the kind of help or assistance that you needed since the outbreak of Covid-19 for you to cope with being in the frontline.
    - What help have you sought to be able to cope and manage the stresses that came with being in the frontline during Covid-19?
    - What keeps you going?
4. **To explore perceptions about COVID-19 vaccines**
    - We now have a vaccine and SA, has the arrival of vaccine changed the environment in the facility (or not)?
        - If yes or no, probe how?
    - How has the take-up of vaccine (or not) been in this facility (by staff and also the patients their seeing). Probe: why do you think that is?
    - Have you been vaccinated yet?
    - If yes, what influenced the decision to vaccinate?
    - If no, please can you share with me the reasons why you are not going to vaccinate?

    - Probe: if it's about information, efficacy, anxiety or other structural reasons for not vaccinating
    - What are your worries and concerns about vaccinating?
    - What do your family think? Are they vaccinated?
    - What do your colleagues think? Are they vaccinated?
  - Please can you share with me what has influenced your decision to not vaccinate?
    - Probe on who, how the decision was influenced and whether there has been shifts over-time
  - What can be done to make you change your mind and go vaccinate?
    - Probe: who needs to do what, and how this can be done?
  - What are your thoughts about considerations of making uptake of COVID-19 vaccine mandatory?

5. **Explore experiences of stigma and discrimination as a frontline health care worker.**
  - How has working in the frontline during the COVID-19 pandemic shaped your experiences of stigma or discrimination?
  - Can you share with me stories where you were stigmatized or discriminated against because of being a frontline worker during the COVID-19 pandemic.
  - Where did it (stigmatization and or discrimination) happen?
    - Within the health facility?
    - In the public spaces (e.g., shopping centres, taxi ranks)
    - At home or in the community
- Would you say the stigmatization or discrimination intensified during specific periods during the pandemic? Please explain.
- Now please tell me about how you responded to experiences of stigmatization or discrimination you experienced because of being a frontline HCW during the COVID-19 pandemic
- How did being stigmatized and or discriminated against because of being a frontline HCWs make you feel?
- Can you share your thoughts on what made some people stigmatized or discriminate frontline HCWs during the COVID-19 pandemic?

Table S2: Themes that emerged from the data

**Level 1**	**Level 2**
Experiences of working in the COVID-19 Ward	Working under pressure with no time to grieve the loss of colleagues
	Complexity of treating COVID-19 patients and feeling less in control
Anxiety and fear of getting infected and death	
Mental health impacts of COVID-19	"I carry the trauma': distress of seeing patients 'dying like flies' everyday
	Symptoms of poor mental health among FHCWs: 'not an easy journey'
Impact on physical health	

Intrinsic motivation and peer support	
Institutional support provided to FHCWs	
